# Soup Consumption Is Associated with a Reduced Risk of Overweight and Obesity but Not Metabolic Syndrome in US Adults: NHANES 2003–2006

**DOI:** 10.1371/journal.pone.0075630

**Published:** 2013-09-30

**Authors:** Yong Zhu, James H. Hollis

**Affiliations:** 1 Department of Epidemiology, University of Iowa, Iowa City, Iowa, United States of America; 2 Department of Food Science and Human Nutrition, Iowa State University, Ames, Iowa, United States of America; University of Catanzaro Magna Graecia, Italy

## Abstract

A limited number of studies have found that soup consumption is related to a lower risk of overweight and obesity in Asian and European populations, however, these studies do not provide a consistent picture regarding the association between soup consumption and markers of metabolic syndrome. To date, no study examining the relationship between soup and body weight or metabolic syndrome have been conducted in the US population. The present study used a sample of 4158 adults aged 19–64 who participated in the National Health and Nutrition Examination Survey between 2003 and 2006. The frequency of soup consumption was determined using a food frequency questionnaire. The weighted prevalence of soup consumption was 94%, with a seasonal variation in the frequency of soup consumption being found. Non-consumers of soup were at a higher risk of being overweight or obese (adjusted odds ratio = 1.381, *P* = 0.013), with a higher adjusted prevalence of reduced HDL cholesterol (adjusted odds ratio = 1.280, *P* = 0.045), but there was no association between soup consumption and metabolic syndrome (*P* = 0.520). The frequency of soup consumption was inversely associated with covariate-adjusted body mass index and waist circumference (*P*<0.05), but not with biomarkers of metabolic syndrome, except for a lower fasting insulin level in frequent soup consumers (*P* = 0.022). Results from the present study suggest soup consumption is not associated with metabolic syndrome. However, there is an inverse relationship between soup consumption and body weight status in US adults, which support laboratory studies showing a potential benefit of soup consumption for body weight management.

## Introduction

The number of people with overweight/obesity or metabolic syndrome has increased over the past few decades [1], 2. Recent studies report that 69.2% of US adults were overweight or obese in 2009–2010 [3], whereas 34% of US adults met the criteria for metabolic syndrome in 2003–2006 [4]. These disorders place economic burdens on both the individual and society [5]. Moreover, they increase the risk of developing other chronic diseases and are associated with increased mortality [6], [7], [8]. Identifying risk factors of overweight/obesity or metabolic syndrome could be helpful for the development of enhanced preventive strategies. To date, a number of dietary factors, such as higher intake of low energy dense foods or dietary fiber, low-fat diets and reduced consumption of sugar-sweetened beverages have been shown a protective effect [9], [10].

Recent laboratory studies have shown that soup served in various forms (such as broth, puree or chunky soup) can reduce appetite or energy intake [11], [12], [13], [14], possibly by delaying gastric emptying and increasing glycemic response [15]. In addition, long-term studies have found a beneficial effect of regular soup consumption on body weight [16], [17] or metabolic profiles [18], [19], [20]. While these trials indicate a potential protective effect of soup consumption on overweight/obesity or metabolic syndrome, only a limited number of studies using a large sample size that is representative of the general population have been conducted [21], [22], [23], [24], [25].

The association of soup consumption and body weight has been studied in a Japanese population [21] and some European countries [22], [23], [24], [25]. Results from these studies consistently report that regular soup consumption is associated with a reduced risk of overweight or obesity [21], [22], [23], [24], [25]. Three of these five studies have also determined the association between soup consumption and some biomarkers related to metabolic syndrome with mixed results reported [21], [22], [23]. Instead of using diagnostic criteria to define whether a participant had metabolic syndrome, these studies have evaluated each biomarker for metabolic syndrome separately [21], [22], [23], as a result, the association between soup consumption and the overall risk of metabolic syndrome remains unknown.

To our knowledge, no epidemiologic studies have been conducted to investigate the association between soup consumption and health status in the US population. The objective of this study was to determine the relationship between soup consumption and body weight status as well as metabolic syndrome in US adults, using data collected in the National Health and Nutrition Examination Survey (NHANES).

## Materials and Methods

The NHANES is a cross-sectional survey designed to assess the health and nutritional status of non-institutionalized residents in the United States. It involves interview and physical examination. In addition, biochemical analyses of blood samples collected from selected subpopulations were performed. Since 1999, the survey examines about 5000 persons each year and the data are released every two years. Its protocol was approved by National Center for Health Statistics Research Ethics Review Board and written informed consent was obtained from all participants; complete details related to sampling methodology, survey instruments, raw data processing, laboratory analysis and quality controls are available on the NHANES website [26].

For the current analysis, data from the 2003–2004 [27] and 2005–2006 [28] survey cycles were combined to maximize the statistical power. 10122 participants participated in the 2003–2004 survey cycle, and 10348 participants were surveyed during 2005–2006. Among a total of 20470 participants, 19593 of them were both interviewed and examined at a mobile examination center, where they completed various questionnaires and measurements. These participants were also asked to provide a 24-h dietary recall. 3–10 days later, a second dietary recall was conducted by telephone. In the 2003–2006 survey cycles, a food frequency questionnaire (FFQ) was also administered for participants older than 2 years who provided at least one dietary recall. The FFQ was developed by National Cancer Institute based on a food frequency instrument that is widely used in nutritional epidemiology research [29].

### Participants

In the present study, adults aged from 19–64 were initially included (n = 7894). Among these participants, 3736 participants were further excluded as they met one or more of the following criteria: invalid or missing response (including participants who were not provided with the FFQ) to the question “did you eat soups over the past 12 months” in the FFQ (n = 3214); pregnant or lactating women (n = 679); or missing values on body weight, height or waist circumference (n = 660). The final sample included 4158 eligible participants.

### Outcome Variables

The response to the question “Did you eat soups over the past 12 months” in the FFQ was used to define the status of soup consumption, with “Yes” for soup consumers and “No” for non-consumers. For soup consumers, the frequency of soup consumption was further assessed by their response to questions “How often did you eat soup during the winter” and “How often did you eat soup during the rest of the year”. For each question, the original responses were categorized into infrequent (less than once per month), moderate (1–3 times per month) and frequent (4 or more times per month) consumers. Participants whose response was “never” for these two questions were also included as non-consumers when the data for the frequency of soup consumption were analyzed.

Body weight and height were measured by trained interviewers using standardized procedures with calibrated equipment. Waist circumference was measured by a soft tape placed horizontally just above the iliac crest. The body weight status was assessed by body mass index (BMI) and categorized to underweight (BMI <18.5 kg/m^2^), normal (BMI 18.5–24.9 kg/m^2^), overweight (BMI 25.0–29.9 kg/m^2^) and obese (BMI ≥30.0 kg/m^2^) using the CDC guideline [30].

Markers for metabolic syndrome included waist circumference, blood pressure as well as metabolites measured from blood samples, including non-fasting HDL-cholesterol, total cholesterol, C-reactive protein, as well as fasting glucose, insulin, triglycerides and LDL-cholesterol 31,32. Metabolic syndrome is defined using the diagnosis guideline from American Heart Association and National Heart, Lung and Blood Institute, that is, meeting any 3 of the 5 criteria: elevated waist circumference (≥102 cm in men, ≥88 cm in women); elevated triglycerides (≥1.7 mmol/L); reduced HDL-cholesterol (<1.03 mmol/L in men, <1.3 mmol/L in women); elevated blood pressure (≥130 mmHg systolic blood pressure or ≥85 mmHg diastolic blood pressure or current use of antihypertensive medications); elevated fasting glucose (≥5.55 mmol/L) pressure or current use of hypoglycemic medications [31]. As NHANES analyzed each metabolite in different subpopulations, the current study only had 1850 participants who did not have any missing values in the five variables related to the above diagnostic criteria for metabolic syndrome.

### Covariates

A number of covariates, including age, gender, race, socio-economic status, physical activity and energy intake were included in the analysis [33], [34]. Participants were recoded into four ethnic groups: Hispanic, non-Hispanic White, non-Hispanic Black and other. To adjust for socio-economic status, the NHANES variable “ratio of family income to poverty” was used and recoded into three groups: high (≥3.50), medium (1.86–3.49) and low (≤1.85) [35]. Physical activity was defined using the response to the question “average level of physical activity each day” from NHANES Physical Activity Questionnaire, as sedentary (sit during the day and do not walk very much), active (stands or walks a lot during the day, but do not have to carry or lift things very often), moderately active (lift light load or have to climb stairs or hills often) or vigorously active (do heavy work or carry heavy loads) [36]. Energy intake was obtained from the 24-h dietary recall. If there were two dietary recalls available, average energy intake was used. In addition, self-reported diabetic status was also included as a covariate for the analysis of fasting glucose and insulin data [32].

### Statistical Analysis

The data were analyzed by SAS version 9.3 (SAS Institute, Cary, NC, USA). To account for the complex multistage design of NHANES, four-year sample weight and specific SAS survey procedures were used in all analyses. Chi-square test was applied as bivariate analyses for a comparison of characteristics between non-consumers and soup consumers. Logistic regression was used to obtain the covariate adjusted odds ratio for overweight/obesity (BMI ≥25 kg/m^2^) and metabolic syndrome according to consumption and non-consumption of soup. In addition, the adjusted odds ratio for having each component of metabolic syndrome was obtained was obtained. Multivariate linear regression was used to compare the covariate-adjusted BMI and waist circumference between non-consumers and soup consumers. It was also used to assess the association between frequency of soup consumption and BMI, waist circumference as well as markers of metabolic syndrome, using non-consumers as the reference group. Observations with missing values were excluded from modeling. Data were presented in weighted percent ± standard error or adjusted least square mean ± standard error where appropriate. Significance was considered as P<0.05. The present secondary analysis only involved publicly available and de-identified data, thus it was exempted from review by institutional review boards at University of Iowa and Iowa State University.

## Results

### Characteristics of Participants

Comparison of participants and non-participants did not reveal any significant difference (data not shown). According to the response to the question “did you eat soups over the past 12 months”, 3816 participants were soup consumers and 342 participants were non-consumers. The weighted percent were 94.0±0.4% and 6.0±0.4%, respectively. Characteristics of participants are shown in Table 1. Compared with non-consumers, soup consumers had a higher percentage of females (*P*<0.001), a higher percentage of non-Hispanic white (*P*<0.001) and a higher percentage of participants who had a higher ratio of family income to poverty (*P*<0.001). In addition, less soup consumers were vigorously active (*P* = 0.001). The bivariate analysis of association between soup consumption and body weight status revealed soup consumers had a higher percentage of people with normal body weight (*P* = 0.041). However, no association between soup consumption and metabolic syndrome was found (*P* = 0.714). When each diagnostic criteria for metabolic syndrome was evaluated, it was found the percentage of people with a reduced HDL cholesterol status was lower in soup consumers (*P = *0.023). Nonetheless, there was no association between soup consumption and conditions of elevated waist circumference, elevated triglycerides, elevated blood pressure or elevated fasting glucose (*P*>0.05).

**Table 1:** Characteristics of participants.

	Non-consumers		Soup consumers		
Characteristics	N^a^	Weighted percent^b^	N^a^	Weighted percent^b^	*P* value^c^
Gender					<.001
Male	197	63.9±3.7	1793	46.5±0.7	
Female	145	36.1±3.7	2023	53.5±0.7	
Race					<.001
Hispanic	79	12.4±2.3	858	10.7±1.2	
Non-Hispanic White	105	55.4±3.8	1975	74.1±2.2	
Non-Hispanic Black	143	25.2±2.8	808	9.6±1.2	
Other	15	7.1±2.0	175	5.5±0.6	
Ratio of income to poverty					<.001
Less or equal to 1.85	184	45.8±3.6	1316	24.8±1.4	
Between 1.85 and 3.50	73	26.5±3.4	899	25.2±1.5	
Greater or equal to 3.50	62	27.6±2.6	1446	50.0±2.2	
Physical activity					0.001
Sedentary	67	20.9±2.5	858	23.3±0.9	
Active	172	46.6±3.2	1942	49.9±1.1	
Moderately active	54	16.7±2.1	686	18.4±0.9	
Vigorously active	49	15.8±2.6	327	8.4±0.6	
Weight status^d^					<0.05
Underweight	13	3.3±1.3	74	1.9±0.3	
Normal	84	25.5±2.6	1203	33.1±1.2	
Overweight	104	33.3±3.3	1220	31.7±1.0	
Obese	141	37.9±3.1	1319	33.3±1.4	
Metabolic syndrome^d^					0.714
Yes	34	23.4±5.0	380	21.6±1.0	
No	113	76.6±5.0	1323	78.4±1.0	
Elevated waist circumference^d^					0.831
Yes	171	48.7±3.6	1906	49.5±1.3	
No	171	51.3±3.6	1910	50.5±1.3	
Elevated triglycerides^d^					0.434
Yes	46	34.1±6.3	490	29.4±1.2	
No	98	65.9±6.3	1158	70.6±1.2	
Reduced HDL cholesterol^d^					<0.05
Yes	109	37.6±3.0	1168	31.5±0.9	
No	206	62.4±3.0	2512	68.5±0.9	
Elevated blood pressure^d^					0.393
Yes	26	6.9±1.8	319	8.6±0.7	
No	300	93.1±1.8	3382	91.4±0.7	
Elevated fasting glucose^d^					0.902
Yes	44	29.6±6.5	518	28.7±1.9	
No	101	70.4±6.5	1146	71.2±1.9	

a Total participants n = 4158. The total N by each specific characteristic may be less than that due to missing values.

b Data were weighted percentage ± standard error.

c Chi-square P value was obtained from bivariate analyses for the association between soup consumption and each specific characteristic.

d Body weight status was based on the CDC guideline (Ref #30); criteria for other conditions were defined by American Heart Association and National Heart, Lung and Blood Institute (Ref #31). See text for details.

### Covariate Adjusted Outcomes and Risk of Overweight/Obesity and Metabolic Syndrome

Covariate adjusted BMI and waist circumference for soup consumers were 28.4±0.2 kg/m^2^ and 95.9±0.5 cm, which were significantly lower than that of non-consumers (29.8±0.6 kg/m^2^, *P* = 0.025; 99.0±1.3 cm, *P* = 0.023). However, there was no difference in covariate adjusted values of triglycerides, fasting glucose, HDL cholesterol and blood pressures between soup consumers and non-consumers (*P*>0.05, data not shown).

Results from logistic regression analyses are summarized in Table 2. Non-consumers were at a higher risk of being overweight or obese (adjusted odds ratio = 1.381, *P* = 0.013). Nonetheless, the adjusted odds ratio for the prevalence of metabolic syndrome [37] was not significant (adjusted odds ratio = 1.218, *P* = 0.520). Each diagnostic criterion for metabolic syndrome was also evaluated. Although there was no effect of soup consumption on the status of fasting glucose, triglycerides or blood pressure (*P*>0.05), compared with soup consumers, non-consumers had a higher adjusted prevalence of elevated waist circumference (adjusted odds ratio = 1.348, *P* = 0.066) as well as reduced HDL cholesterol (adjusted odds ratio = 1.280, *P* = 0.045).

**Table 2:** Adjusted odds ratio of having certain conditions related to body weight status and metabolic syndrome and its 95% confidence interval: non-consumers vs soup consumers in NHANES 2003–2006.

Conditions^a^	N^b^	Adjustedodds ratio^c^	95% CI	*P* value
Overweight/Obesity	3977	1.381	1.070–1.783	<0.05
Metabolic syndrome	1761	1.218	0.669–2.218	0.520
Elevated waistcircumference	3977	1.348	0.981–1.851	0.066
Elevated triglycerides	1822	1.185	0.694–2.023	0.534
Elevated fasting glucose	1833	1.262	0.616–2.583	0.525
Reduced HDL cholesterol	3824	1.280	1.005–1.630	<0.05
Elevated blood pressure	3851	0.859	0.504–1.466	0.578

a Overweight/Obesity is defined by BMI≥25.0 kg/m^2^; criteria for other conditions were defined by American Heart Association and National Heart, Lung and Blood Institute guideline (ref #31). See text for details.

b Sample size used in the model. They were less than 4158 because of missing values.

c Logistic regression models adjusting for age, gender, race, poverty income ratio, physical activity and energy intake.

### Frequency of Soup Consumption and Body Weight, Markers of Metabolic Syndrome

The frequency of soup consumption is shown in Table 3. A seasonal variation was observed. For example, 40.7% of participants were frequent soup consumers whereas 27.3% were infrequent soup consumers in winter. In other seasons, the percentages were 18.6% and 39.0%, respectively.

**Table 3:** Frequency of soup consumption in US adults aged 19–64: NHANES 2003–2006.

	Consume soup during the winter?		Consume soup during the rest of the year?	
	N	Weighted percent^a^	N	Weighted percent^a^
Non-consumers^b^	353	6.1±0.4	522	10.3±0.6
Infrequent (<1 time per month)	1157	27.3±0.9	1571	39.0±1.2
Moderate (1–3 times per month)	1057	25.9±0.8	1262	32.1±1.2
Frequent (≥4 times per month)	1578	40.7±1.1	787	18.6±0.9
Total^c^	4145	100.0	4142	100.0

a Data were weighted percentage ± standard error.

b Non-consumers also included those participants whose response was “No” for the previous question “did you eat soups over the past 12 months?”.

c The total N was not equal to 4158 because of missing responses.

The frequency of soup consumption was inversely associated with BMI and waist circumference after controlling for age, gender, race, socio-economic status, physical activity and energy intake, and this was independent to the season when soup was consumed (Table 4). A linear decrease in both BMI and waist circumference was observed as the frequency of soup consumption increased. In addition, it was found that frequent soup consumption during the rest of the year was associated with a lower fasting insulin level (*P* = 0.022), however such a reduction in fasting insulin level did not reach the significance level when data for winter soup consumption was analyzed (*P* = 0.103). Moreover, there was no association between soup consumption and other markers of metabolic syndrome (Table 4).

**Table 4:** Adjusted least square mean of body mass index, biomarkers of metabolic syndrome by frequency of soup consumption: NHANES 2003–2006.

	Consume soup during the winter?		Consume soup during the rest of the year?	
	LS mean±SE^a^	*P* value^b^	LS mean±SE^a^	*P* value^b^
BMI (kg/m^2^)				
Non-consumers^c^	29.8±0.6	Ref.	29.5±0.4	Ref.
Infrequent (<1 time per month)	28.6±0.3	0.063	28.6±0.3	<0.05
Moderate (1–3 times per month)	28.3±0.2	<0.05	28.2±0.3	<0.05
Frequent (≥4 times per month)	28.4±0.3	<0.05	28.3±0.4	<0.05
Waist Circumference (cm)				
Non-consumers^c^	99.0±1.3	Ref.	98.4±1.0	Ref.
Infrequent (<1 time per month)	96.5±0.8	0.098	96.4±0.6	<0.05
Moderate (1–3 times per month)	95.6±0.6	<0.05	95.4±0.7	<0.05
Frequent (≥4 times per month)	95.7±0.7	<0.05	95.4±0.8	<0.01
Glucose (mmol/L)^d^				
Non-consumers^c^	5.64±0.11	Ref.	5.60±0.08	Ref.
Infrequent (<1 time per month)	5.57±0.07	0.549	5.58±0.08	0.749
Moderate (1–3 times per month)	5.61±0.11	0.790	5.59±0.09	0.900
Frequent (≥4 times per month)	5.56±0.07	0.483	5.55±0.07	0.559
Insulin (pmol/L)^d^				
Non-consumers^c^	80.7±10.3	Ref.	77.5±5.6	Ref.
Infrequent (<1 time per month)	64.7±3.3	0.158	67.2±3.8	0.075
Moderate (1–3 times per month)	69.3±5.1	0.349	63.7±4.4	0.101
Frequent (≥4 times per month)	62.2±2.9	0.103	62.2±4.1	<0.05
C-reactive protein (mg/L)^e^				
Non-consumers^c^	4.0±0.5	Ref.	3.8±0.3	Ref.
Infrequent (<1 time per month)	4.4±0.3	0.438	4.2±0.3	0.944
Moderate (1–3 times per month)	3.9±0.3	0.934	4.3±0.3	0.246
Frequent (≥4 times per month)	4.0±0.3	0.937	3.7±0.5	0.331
Total cholesterol (mmol/L)^e^				
Non-consumers^c^	5.07±0.07	Ref.	5.09±0.06	Ref.
Infrequent (<1 time per month)	5.14±0.05	0.411	5.12±0.04	0.628
Moderate (1–3 times per month)	5.12±0.03	0.534	5.16±0.04	0.249
Frequent (≥4 times per month)	5.12±0.04	0.469	5.10±0.05	0.831
HDL cholesterol (mmol/L)^e^				
Non-consumers^c^	1.37±0.03	Ref.	1.36±0.02	Ref.
Infrequent (<1 time per month)	1.38±0.02	0.670	1.40±0.02	0.121
Moderate (1–3 times per month)	1.42±0.02	0.126	1.40±0.01	0.128
Frequent (≥4 times per month)	1.40±0.02	0.252	1.40±0.02	0.110
LDL cholesterol (mmol/L)				
Non-consumers^c^	2.91±0.09	Ref.	2.93±0.10	Ref.
Infrequent (<1 time per month)	3.01±0.06	0.282	2.98±0.04	0.565
Moderate (1–3 times per month)	2.92±0.05	0.932	2.92±0.06	0.929
Frequent (≥4 times per month)	2.88±0.05	0.765	2.87±0.05	0.562
Triglycerides (mmol/L)				
Non-consumers^c^	1.76±0.24	Ref.	1.64±0.14	Ref.
Infrequent (<1 time per month)	1.53±0.06	0.367	1.52±0.06	0.385
Moderate (1–3 times per month)	1.49±0.07	0.305	1.49±0.06	0.260
Frequent (≥4 times per month)	1.48±0.06	0.271	1.48±0.09	0.301
Systolic blood pressure (mmHg)				
Non-consumers^c^	122±1.1	Ref.	121±1.1	Ref.
Infrequent (<1 time per month)	121±0.6	0.311	122±0.6	0.745
Moderate (1–3 times per month)	122±0.7	0.724	121±0.8	0.895
Frequent (≥4 times per month)	121±0.8	0.468	121±0.8	0.916
Diastolic blood pressure (mmHg)				
Non-consumers^c^	72±0.9	Ref.	72±0.6	Ref.
Infrequent (<1 time per month)	72±0.5	0.913	73±0.5	0.302
Moderate (1–3 times per month)	72±0.5	0.488	72±0.6	0.950
Frequent (≥4 times per month)	72±0.5	0.897	72±0.7	0.829

a Data were expressed as the least square mean ± standard error, adjusted for age, gender, race, poverty income ratio, physical activity and energy intake.

b P values for comparisons using non-consumers as the reference group.

c Non-consumers also included those participants whose response was “No” for the previous question “did you eat soups over the past 12 months?”.

d Values were also adjusted for self-reported diabetic status in addition to other covariates.

e Total cholesterol, HDL cholesterol and C-reactive protein were analyzed in non-fasting samples by NHANES.

## Discussion

The present analysis revealed that 94% of US adults aged 19–64 were soup consumers and there was a seasonal variation in the frequency of soup consumption. Similar variation has been reported by Bertrais *et al.* [23], who found that soup consumption was higher in autumn and winter, with a maximum in January in a French population. While data from the present study suggest the soup consumption was common in US, the frequency of soup consumption was lower compared with other countries. For example, approximately 60% of US adults consume soup less than four times per month (or less than once per week) in winter. This number increased to over 80% during other seasons. In Japan, the median of soup consumption was 7 times per week [21], which indicated over half of the population was daily soup consumers. The study in France had also shown that 46% women and 42% men were regular consumers who ate soup 3–4 times per week [23]. Although different periods of dietary history (such as soup consumption over past month or past year) were assessed in these studies [21], [23], which could partly account for the difference in the frequency of soup consumption, it is much possible that the difference among these countries were due to different dietary habits in different cultures.

Results from the present analysis indicated that soup consumption was associated with a lower BMI and waist circumference, as well as a reduced risk of being overweight or obese in the US adults after controlling for possible confounders. This is consistent with results from previous studies in Asian and European countries [21], [22], [23], [24], [25]. Giacosa *et al.* [22] had conducted a study in Italy and found the prevalence of obesity in adults was lower in soup eaters (4%), compared with that in non-consumers (13%). In France, a higher frequency of BMI <23 kg/m^2^ in heavy soup consumers (5–6 times per week) was found, with a higher frequency of BMI >27 kg/m^2^ in occasional and non-soup consumers being reported [23]. The association of soup consumption and body weight in Portuguese population were evaluated in children [25] and adults [24], with both studies suggesting a reduced risk of obesity in soup consumers [24], [25]. In addition, an inverse association between the frequency of soup consumption and BMI as well as waist circumference was reported in Japan [21]. Although all these studies [21], [22], [23], [24], [25] used different study design with sample sizes varied from 103 to nearly 40000, in view of their results, the inverse association between soup consumption and body weight status appears to be consistent across different countries.

Our study revealed non-consumers were at a higher risk for reduced HDL cholesterol. In addition, frequent soup consumption was associated with a lower fasting insulin level. However, soup consumption was not associated with the overall risk of metabolic syndrome and it did not impact other biomarkers of metabolic syndrome. In this study, because biomarkers of metabolic syndrome were analyzed in different subgroups by NHANES, the status of metabolic syndrome was only known for 1850 participants. This may have limited the power of the study. Currently only a few studies have investigated the association of soup consumption and biomarkers of metabolic syndrome. Giacosa *et al.* [22] had shown soup eaters had a beneficial profile on cholesterol, triglycerides and blood pressure, whereas Bertrais *et al.* [23] found there was a lower incidence of hypercholesterolemia in heavy soup consumers. By contrast, Kuroda *et al.* [21] failed to observe such associations between soup consumption and any metabolic biomarkers. These inconsistent results may be explained, in part, by the variation in sample size and the difference in characteristics of participants, such as genetic background and dietary habits.

The association between soup consumption and body weight observed in the present study does not imply any casual effect. Nonetheless, it supports previous laboratory interventions that have found a beneficial effect of soup consumption on body weight. Jordan *et al.* [16] conducted a study to evaluate a 10-week behavioral program for weight loss and found participants who ate soup four or more times a week lost 20.4% for their excess weight (defined as the difference between actual weight and ideal weight), by contrast, those who ate soup less than four times a week lost only 14.7% of their excess weight. Another study had also evaluated the effect of soup consumption in addition to a traditional weight loss program [17]. It was found the average number of cups of soup per day was highly correlated with weight loss, moreover, participants in the soup group maintained their weight loss better than participants in the traditional group at the follow-up 52 weeks later [17]. Nonetheless, the mechanism that explains the observed effects remains unclear. One of the potential reasons could be that energy density of soup may be lower due to its large amount of liquid content. Consumption of low energy dense food is associated with reduced appetite or food intake [38] and a lower risk for obesity [39], [40]. While recent studies suggest the satiating effects of soup [11], [12], [13], [14], change in postprandial appetite may not be accompanied by reduced food intake at the subsequent meal or the satiating effects may be short-lived [41], [42]. It remains unclear whether the short-term change in appetite in response to a soup preload will be compensated later, and how it contributes to long-term energy homeostasis.

There are other limitations to this study. First, the FFQ was not quantitative, as a result, the amount of soup consumption was not known. Even though frequency of soup consumption was reported, it might not be correlated with the amount of soup consumption, due to potential variations in the amount consumed each time. Second, it suffers from a potential report bias as soup consumption over past 12 months was recalled. Participants might not be able to recall their diet history accurately. In addition, some biomarkers were measured in non-fasting samples by NHANES. Like other studies [32], [33] that have evaluated risk factors for obesity and metabolic syndrome, health status and medications were not used as exclusion criteria or covariates in this study, to increase generalizability and avoid potential overlap between these variables and definition of outcome variables. Those factors could influence biomarkers examined in this study. Despite these limitations, the study consists of a large sample size representing the general US adults. To our knowledge, it was the first study that had found the soup consumption was associated with a reduced risk of overweight or obesity in US population. In addition, it was noted that the frequency of soup consumption was much lower than that in Asian and European countries. As a result, increasing the frequency of soup consumption is recommended.
